# A Recent Lecture Outlined
*Presidential address delivered before the Society of Tropical Medicine and Hygiene on Friday, October 17, by Dr. W. Simpson.


**Published:** 1919-12-06

**Authors:** 


### MODERN VIEWS ON PREVENTABLE DISEASE.

### A Recent Lecture Outlined.*

### Theories of the Ancients.

Our ancestors attributed outbreaks of disease to an epidemic constitution of the air and its influence on man's economy. That epidemic constitution has still to be studied. It has been lost sight of in recent years, though earlier it engaged the attention of philosophers who ascribed the origin and exceptional spread of epidemics to unusual meteorological conditions which caused floods, drought, and famine. Who shall prove that they were wrong? What effect have floods, drought, and famine on the parasites of disease or on the various animal and vegetable micro-organisms that teem in the soil? Do parasites gain virulence and increased powers of diffusion in the soil, or in the diseased vegetable world subjected to these conditions, or in the herbivorous animals that subsist on it, or in the vermin and insects that feed on the diseased animals and plants, or in man who, when famished, is glad to eat any vegetable, seed, grass, fruit, or meat, even if diseased? Apart from the virulence and diffusiveness gained by the sojourn of the microbe in one or several of these media, what are the factors which combine to form an endemic area for a predominant microbe? Many such problems are but partially solved.

### Geological Considerations.

The geological nature of the soil often limits the geographical distribution and kinds of animals existing underground. Perhaps it is in this direction that the explanation may be found for the original endemic sites of cholera, plague, influenza, yellow fever, and cerebro-spinal disease. Perhaps Cunningham, of Calcutta, was on the correct path when investigating the nature and composition of the soil and its gaseous, organic, and inorganic contents. Possibly it is the interaction of unusual meteorological conditions on the local fauna and flora of certain limited area, that prepares the micro-organisms for their migration beyond their endemic homes—not by the air, as Bryden and others used to think of cholera, but by making use of man, the lower animals and insects, and sometimes inanimate objects, as carriers.

### The Need for Future Knowledge.

It is necessary to know more about the endemic areas of disease: they should be sought out and classified. It is there that the natural history of disease in man can best be studied in relation to the geology, fauna, flora, and other conditions existing in the locality, and it is there that the conditions have to be investigated to give us a clue to the factors which convert a sporadic or endemic non-spreading or self-limiting disease into a diffusive and wide-spreading epidemic or pandemic. This knowledge will allow us to develop successful measures at the very fountain head,

Although much has been discovered in regard to preventable disease, there are still vast problems awaiting solution by research. We require to know more about plague, cholera, smallpox, leprosy, influenza, cerebro-spinal fever, trypanosomiases, yellow fever, blackwater fever, and many other tropical diseases and their relation to man's environment, not only in his home, but also in nature. Where these diseases commonly prevail research laboratories are necessary, well-equipped with all the modern resources which bacteriology, protozoology, parisitology, biochemistry, and geology can place at our disposal.

### The Dissemination of Disease in Peace and War.

War and epidemics bring out the weak points of our defence against disease, but their after-effect is generally to set men's minds actively in train to devise means for future protection and ideas and schemes that have for many years only created a languid interest get energised and become reality. This is seen in the recent creation of a Ministry of Health in this country, which has been advocated for fifty years, but which the war and epidemic of influenza has quickly brought into being. It was the Boer War, with its enormous number of dysentery and typhoid cases, that led to the reinstatement of the sanitary officer in the British Army.

### Disease and the Navy.

The British Navy led the way in protecting her fleets against disease in the tropics. During the wars with France and Spain in 1778 and the following years the West Indies was the principal seat of naval operations, and much greater fleets were employed in that quarter of the world than in any former period. There was much sickness in the fleet, varying greatly in different ships, and consisting of dysentery, scurvy, and fever. Dr. Blane (afterwards Sir Gilbert Blane), when Physician to the Fleet under Lord Rodney, had monthly returns sent to headquarters recording the sickness and deaths in each ship, and anything else which related to the health of the crew. He found that the average mortality in 12 months was 1 in 7 of the seamen, and on an average 1 man in 15 was on the sick list. In 1781 he addressed a memorial to the Board of Admiralty pointing out the excessive mortality, and expressing his conviction that if certain prosposals which he suggested were carried out, more than two-thirds of the seamen who died in that climate would be saved, and we know how various sanitary regulations were later enforced, and how their result was seen in the remarkable transition from the 1 in 7 mortality of 1781 to 1 in 310 of the present time.

### The Tendency to Forget Past Lessons.

We are apt to forget the lessons of the past. For instance, in the Mesopotamian campaign there would

* Presidential address delivered before the Society of Tropical Medicine and Hygiene on Friday, October 17, by Dr. W. Simpson.

**Modern Views on Preventable Disease** - (*continued*).

have been no scurvy if the history of the Navy in relation to that disease, and its abolition in the latter part of the eighteenth century, had been borne in mind and acted upon.

One often wonders whether the many relapses of the soldiers from Salonika and other seats of war were due to our preference for quinine, for which there has been an unprecedented demand and some samples of which may have in their preparation lost the complete qualities of the cinchona bark, which itself varies in quality. Are we sure that the procedure which destroys the vitamines of canned food may not also affect some of the properties of the alkaloids?

The author described the beginnings and progress of sanitary organisation in India, and deplores the Lilliputian schemes of the Indian Government. He laid great emphasis upon the need for education as to the needs of sanitary science, and concluded his address by a strong appeal:—

SCHEME FOR AN EMPIRE HEALTH MUSEUM.

"When H.M. King Edward VII. died, I wrote to the Lord Mayor of London, who was Chairman of the Committee, to consider a suitable memorial to commemorate his reign. I ventured to suggest an Imperial Health Institute for Research with an Empire Health Museum, on a similar or larger scale than that of the British Museum. My suggestion was not accepted at the time.

"Now that there will be an Empire War Memorial worthy of the far-reaching victory which has gained safety for us all, I would suggest that in addition to the War Memorial there should be a Peace Memorial in the form of an Empire Health Museum in London, in every respect to celebrate the means by which we can triumph over preventable diseases, which, in the long run, are far more destructive to human life and to health than even this great war. The Museum would be a standing reminder to our administration that, next to the safety of the Empire is the health of the people."

